# Internet use of parents before attending a general pediatric outpatient clinic: does it change their information level and assessment of acute diseases?

**DOI:** 10.1186/s12887-016-0677-8

**Published:** 2016-08-18

**Authors:** Christian Sebelefsky, Jasmin Voitl, Denise Karner, Frederic Klein, Peter Voitl, Andreas Böck

**Affiliations:** 1 First Vienna Pediatric Medical Center, Donau-City-Straße 1, 1220 Wien, Austria; 2 Department of Pediatrics and Adolescent Medicine, Medical University of Vienna, Währinger Gürtel 18-20, 1090 Wien, Austria; 3 Independent Statistician, Augsburg, Germany

**Keywords:** Internet child health information, Information level, Assessment of acute diseases, Online resources, Influencing factors, Parents

## Abstract

**Background:**

Before seeing a pediatrician, parents often look online to obtain child health information. We aimed to determine the influence of IUC (internet use regarding the reason for consultation) on their subjective information level, their assessment of acute diseases and the change in this assessment. Secondary objectives were to identify the most commonly used online resources and factors with an influence on IUC.

**Methods:**

This cross-sectional observational study was conducted at a general pediatric outpatient clinic located in Vienna, Austria. An anonymous, voluntary and 14-items-containing questionnaire served to gather all data. A total number of 500 questionnaires were collected.

**Results:**

Of the parents attending the outpatient clinic, 21 % use the internet before the appointment (= IUC). Most common online resources utilized for this purpose are websites run by doctors (61.3 %), the outpatient clinic’s homepage (56.3 %), Google (40 %), Wikipedia (32.5 %), health advisory services provided by doctors (28.7 %), health portals (21.3 %) and health forums and communities (18.8 %). The information level in terms of the reason for consultation is rated as good by 50.6 %, as average by 46.7 % and as insufficient by 2.7 % (internet users: 42.7 %, 55.3 %, 1.9 %). Acute diseases of the children are estimated to be mild by 58.4 %, to be moderate by 41.1 % and to be severe by 0.5 % (internet users: 54.9 %, 45.1 %, 0 %). After having used any source of information, this assessment is unchanged in 82.8 %, acute diseases are rated as more severe in 13.8 % and as less severe in 3.4 % (internet users: 79.2 %, 16.7 %, 4.2 %). Internet users and non-users do not differ with respect to their information level (*p =* 0.178), the assessment of acute diseases (*p =* 0.691) and the change in this assessment (*p =* 0.999). A higher education level of parents (mothers: *p =* 0.025, fathers: *p =* 0.037), a young age of their children (*p =* 0.012) and acute diseases of their children (*p =* 0.046) predispose to IUC.

**Conclusions:**

Against the common perception that online health information might fuel panic-mongering, we could not determine a link between IUC and the assessment of acute diseases. The information level of internet users and non-users does not differ either. Further research is needed to clarify causes for high and low IUC.

## Background

To date, many studies worldwide have focused on the proportion of parents using the internet to obtain child health information (CHI). It is incontrovertible that the vast majority use the web for this purpose [1–12]. In this article, we make a distinction between general use (general internet use to obtain child health information *=* IUG) and the use before an appointment at a healthcare facility (internet use to be informed about the reason for consultation *=* IUC). This differentiation appears to be necessary in terms of a more comprehensive reflection on the internet health seeking behavior of parents.

We previously reported that, before attending a general pediatric outpatient clinic, more than one in five parents (21 %) use the internet to obtain child health information (IUC). This is most likely the case, if their children present with an acute disease [9]. Nevertheless, it still remains unknown which other factors exert an influence on this specific behavior (influencing factors = IF) and which are the most commonly used child health information resources (internet resources = IR). In addition, it is also unknown which non-web-based sources of information are relevant to parents (other resources = OR). These aspects are worth investigating, especially in case of parents attending with an acutely ill child.

Moreover, the quality of web contents is a major concern. Many previous investigations have shown how parents perceive the usefulness and trustworthiness of child health websites and how medical professionals assess these [2, 3, 8, 10, 12–17]. The knowledge of parents regarding high and low quality web contents and the confidence in using web-based information to make health decisions have also been investigated previously [18, 19]. However, it is still unclear how parents rate their information level with respect to child health issues and if this knowledge is dependent on the use of internet and non-internet CHI. In addition, it seems worth knowing how parents assess the severity of acute diseases of their children and the change in this assessment after using web-based or non-web-based child health information resources. This seems particularly worth knowing, as it is often said of online health seeking to involve the risk of panic-fueling. Health literacy might also play an important role in this context, suggesting the influence of the educational level of parents to be studied in detail. To our knowledge, so far no studies have addressed these specific topics.

### Objectives

The primary objectives of this investigation were to determine:

1. how parents estimate their information level in terms of the reason for consultation (information level = IL)
2. if there are differences between users and non-users of the internet, the most common IR and OR with respect to their information level
3. how parents rate the severity of acute diseases of their children (disease severity assessment = DA)
4. if there are differences in the assessment of acute diseases between users and non-users of the internet, the most common IR and OR
5. if parents experience a change in the assessment of acute diseases (change in disease severity assessment = CA) when using web-based or other sources of information before attending the pediatric outpatient clinic
6. if this change in the rating of acute diseases is contingent on the use of the internet, the most common IR or OR

The secondary objectives of this investigation were to ascertain:

1. if connections exist between the education level of parents and IL, DA and CA
2. the proportions of parents using any source of information, web-based (=IUC) (see also [9]) and non-web-based sources before the appointment (with an additional focus on parents attending with an acutely ill child)
3. the most common IR (with an additional focus on parents attending with an acutely ill child)
4. the most common OR (with an additional focus on parents attending with an acutely ill child)
5. factors that exert an influence on IUC (IF)

## Methods

### Study design and data collection

The cross-sectional observational study underlying this publication was conducted at the First Vienna Pediatric Medical Center (FVPMC), a general pediatric outpatient clinic located in Vienna, Austria. An anonymous, voluntary and 14-items-containing questionnaire in German language (Table 1) served to gather all data. Twelve of these items had to be answered by an accompanying parent, while being at the waiting room. Only one parent per family, volunteering to participate in the study, was allowed to complete the questionnaire. In case of more than one child per family requiring medical attention, parents were advised to pick one in order to answer the questions correctly. Another two items had to be answered by the treating doctor, immediately after the families had left the treatment room. The questionnaire was designed for this study and has not been validated prior to its use, as no standardized instrument is available to survey the internet health seeking behavior of parents. Several investigations have been previously conducted at the FVPMC, which involved questionnaires including items 3–6, 13, and 14 (Table 1). Items 1, 2, and 7–12 were developed exclusively for data collection within this study (Table 1). No definitions of the answer options ‘good’, ‘average’, and ‘insufficient’ (item 10) were given on the survey. This also applies to the answer options ‘mild disease’, ‘moderate disease’, and ‘severe disease’ (item 11). These answer options were subjectively assessed on the part of parents. A sample size of 500 was chosen in order to meet the criteria suggested by Israel [20] without difficulties. In a huge statistical population the data sample should include a minimum of 400 cases (for continuous variables, even less for categorical variables) to keep the sampling error ±5 % [20]. A number of 553 questionnaires were distributed. Fifty-three surplus copies were needed to replace incomplete questionnaires where several items were missing or could not be evaluated. The data collection period lasted from 25 October to 8 November 2013. An average number of 152 pediatric patients (2014) visiting the outpatient clinic with their parents per day (weekdays and weekends) made it possible to keep this period relatively short. Of the parents asked to complete a questionnaire, approximately 10 % declined to participate in the study. All parents and legal guardians (aged 18 and above) of pediatric patients (aged 0 – 17) as well as all kinds of consultations and diseases could be included in the investigation. Parents and legal guardians with insufficient German language skills were excluded.

**Table 1:** Questionnaire items

	Variables (dimensions)	Values (answer options)	Descriptive statistical data
1.	Accompanying parent (completing the questionnaire)	Mother/Father	Mothers: 81.5 % (378/464)
			Fathers: 18.5 % (86/464)
2.	Age of the accompanying parent (completing the questionnaire)	_____ (years)	*n =* 464
			Average: 34 years
			Std. dev.: 6.4 years
			Min.: 18 years
			Max.: 56 years
3.	Highest completed level of education (mother)	Compulsory school/	Compulsory school: 2.8 % (14/500)
		Apprenticeship or technical college/	Apprenticeship or technical college: 30.8 % (154/500)
		High-school diploma/	High-school diploma: 27.6 % (138/500)
		University	University: 38.8 % (194/500)
4.	Highest completed level of education (father)	Compulsory school/	Compulsory school: 5.5 % (27/492)
		Apprenticeship or technical college/	Apprenticeship or technical college: 35.4 % (174/492)
		High-school diploma/	High-school diploma: 24.0 % (118/492)
		University	University: 35.2 % (173/492)
5.	Sex of the child	Female/Male	Girls: 45.8 % (228/498)
			Boys: 54.2 % (270/498)
6.	Age of the child	_____ (years)	*n =* 497
			Average: 2.4 years
			Std. dev.: 2.6 years
			Min.: 0 years
			Max.: 17 years
7.	Internet use to be informed about the reason for consultation (IUC)	Yes/No	Yes: 21.0 % (105/499)
			No: 79.0 % (394/499)
8.	Internet child health information resources (IR)	*For answer options see Fig.* *2* *.* *All options were to be answered with Yes/No.* *Multiple answers (IR) were possible.*	*For descriptive statistical data see Fig.* *2* *.*
9.	Other child health information resources (OR)	*For answer options see Fig.* *3* *.* *All options were to be answered with Yes / No.* *Multiple answers (OR) were possible.*	*For descriptive statistical data see Fig.* *3* *.*
10.	Information level regarding the reason for consultation (IL) (subjective)	Good/Average/Insufficient	*For descriptive statistical data see Fig.* *1* *.*
11.	Disease severity assessment (DA) (subjective)	Mild disease/ Moderate disease/ Severe disease	*For descriptive statistical data see Fig.* *1* *.*
12.	Change in disease severity assessment (CA) (subjective)	Unchanged/ Disease is now rated as more severe/Disease is now rated as less severe	*For descriptive statistical data see Fig.* *1* *.*
13.	Reason for consultation (indicated by the treating doctor)		*n =* 498 (518 responses)
		Acute disease/	Acute disease: 55.4 % (276/498)
		Follow-up visit after an acute disease/	Follow-up visit after an acute disease: 8.4 % (42/498)
		Monitoring of a chronic disease/	Monitoring of a chronic disease: 2.8 % (14/498)
		Preventative check-up/	Preventative check-up: 22.1 % (110/498)
		Vaccination/	Vaccination: 13.5 % (67/498)
		Other reason: _____	Other reason: 1.8 % (9/498)
		*All options were to be answered with Yes/No.* *Multiple answers were possible.*	
14.	Diagnosis/-es (according to ICD-10) (indicated by the treating doctor)	*This item was only evaluated for acute diseases of pediatric patients.*	*This item was only evaluated for acute diseases of pediatric patients.*
		*For subgroups of acute diseases see Fig.* *4* *.*	*For descriptive statistical data see Fig.* *4* *.*
		*All options were to be answered with Yes/No.*	*The following subgroups of acute diseases were excluded from Fig.* *4* *, as these were not chosen: Neoplasms/Diseases of the blood and blood-forming organs and certain disorders involving the immune mechanism/Endocrine, nutritional and metabolic diseases/Diseases of the nervous system/Diseases of the circulatory system/Congenital malformations, deformations, and chromosomal abnormalities/Symptoms, signs, and abnormal clinical and laboratory findings, not elsewhere classified/*
		*Some diagnosis groups (ICD-10) were subdivided into more specific entities and were also to be answered with Yes/No.*	
		*Multiple answers were possible.*	
			*Certain conditions originating in the perinatal period.*

### Statistical methods

After collection of the questionnaires, statistical analysis was done with IBM SPSS Statistics (Version 22.0). For each item frequencies were calculated. All variables with their respective values and descriptive statistical data are shown in Table 1. For statistical testing the confidence interval was set to 95 %.

For reasons of conclusiveness, the seven most common IR (Fig. 2) and the three most common OR (Fig. 3) were selected for statistical testing. Multivariate analysis was applied to examine the influence of IUC and the three most common OR (all nominally scaled, dichotomous) on IL, DA and CA. In order to test for multicollinearity of IUC and the three most common OR with respect to IL, DA and CA, tolerance values of the linear regression model were utilized. Prior to these operations, IL, DA and CA (all three ordinally scaled) were recoded into nominal (dichotomous) variables (IL: ‘Good + Average’/’Insufficient’, DA: ‘Mild + Moderate disease’/’Severe disease’, CA: ‘Change’/’No Change’), as the frequencies of at least one category of each of these were very low (2 ≤ n ≤ 13). Application of the logit model (logistic regression) allowed us to examine the connections between IUC and IL, DA and CA, while controlling the effect of using the three most common OR and vice versa. However, this model was not utilized to determine the effects of using the seven most common IR in this respect. A great number of CHI resources, with partly small numbers of users, would have needed to be considered and might have resulted in impractical models.

The Mann–Whitney *U* test was therefore used to determine if significant statistical differences exist between parents who used and did not use the most common IR (all nominally scaled, dichotomous) regarding IL, DA, and CA (all three ordinally scaled).

The Mann–Whitney *U* test also served to ascertain whether there are differences between internet users and non-users (IUC) (nominally scaled, dichotomous) with respect to their education level (ordinally scaled).

The Spearman correlation and Spearmanʼs rank correlation coefficient (Spearmanʼs rho (ρ)) served to test for significant statistical connections between the education level of parents and IL, DA and CA (all ordinally scaled).

Fisher’s exact test was used in order to identify significant statistical connections between IUC and the sex of parents, the sex of children, the reasons for consultation indicated by the treating doctors, specific subgroups of acute illnesses, and the use of the three most common OR (all nominally scaled, dichotomous). The remaining OR were not used for statistical testing as the numbers of users were too low to produce conclusive results (2 ≤ *n* ≤ 8) (Fig. 3)).

The *t*-test served to analyze significant statistical differences in IUC (nominally scaled, dichotomous) with respect to the age of parents and children (both metrically scaled).

## Results

We previously reported on IUC and the related descriptive results (Table 1) as well as the connection between internet use and acute diseases [9]. For the purpose of a comprehensive reflection, in the following section these results are referred to where appropriate. However, the variable IUC was used for further statistical testing exclusively within this article. Other variables (sex and age of parents and children, education level of parents, reason for consultation and diagnosis) with descriptive results were also presented previously [9].

### Information level regarding the reason for consultation (IL), disease severity assessment (DA) and change in disease severity assessment (CA)

**Fig. 1:**
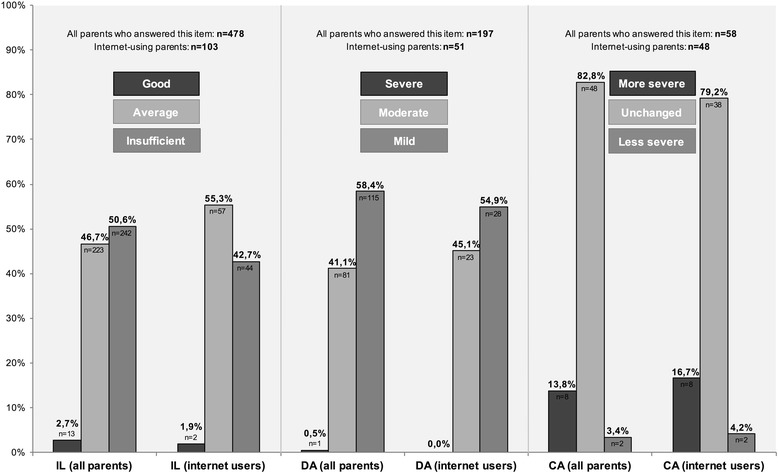
Information level regarding the reason for consultation (IL), disease severity assessment (DA) and change in disease severity assessment (CA) with percentages of answers and numbers of cases (presented separately for all parents who answered this item and internet-using parents)

**Fig. 2:**
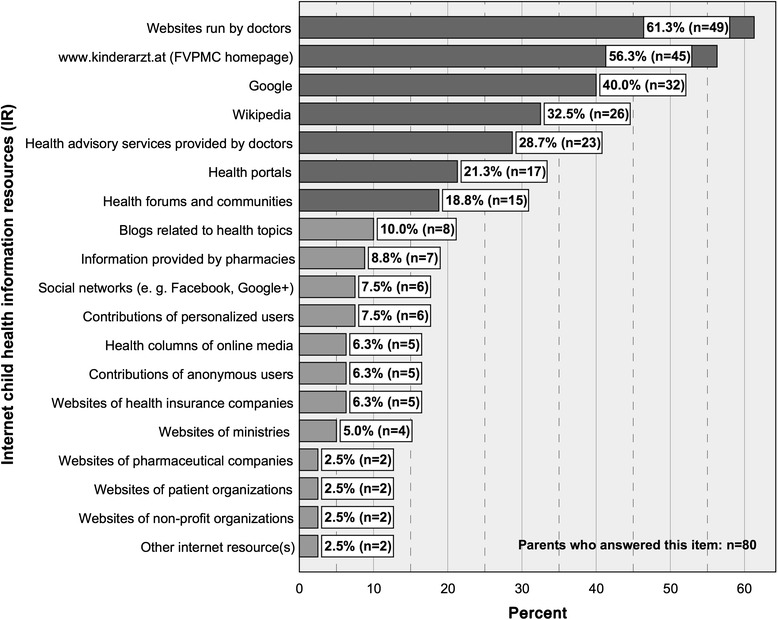
Internet child health information resources (IR) with percentages of users and numbers of cases. FVPMC: First Vienna Pediatric Medical Center

The vast majority of parents indicated to have a good (50.6 %/*n =* 242) or average (46.7 %/*n =* 223) information level regarding the reason for their visit to the pediatrician. It was only stated by 2.7 % (*n =* 13) to have insufficient knowledge (Fig. 1). For internet users the proportions are 42.7 % (good, *n =* 44), 55.3 % (average, *n =* 57) and 1.9 % (insufficient, *n =* 2) (Fig. 1). There is no statistically significant difference between parents who used the internet and those who did not use it in terms of IL (*p =* 0.178). This is also the case for the seven most common IR (Fig. 2) (0.356 ≤ *p ≤* 0.903) and non-internet CHI resources (family member or friend: *p =* 0.207, doctor: *p =* 0.116, pharmacist: *p =* 0.766). The education level of mothers has an influence on their IL (*p =* 0.024), which is not the case for fathers (*p =* 0.971). The higher the education level of mothers, the higher their estimation of their own knowledge in terms of the reason for consultation. Less educated mothers, on the contrary, tend to rate their information level as inferior.

Most of the parents visiting the out-patient clinic with an acutely ill child indicate the disease to be mild (58.4 %/*n =* 115) or moderate (41.1 %/*n =* 81). Merely one parent (0.5 %) rated the acute disease of their child to be severe (Fig. 1). For internet users the proportions are 54.9 % (mild, *n =* 28), 45.1 % (moderate, *n =* 23) and 0 % (severe) (Fig. 1). The childʼs disease severity is not assessed differently by internet users compared to non-users (*p =* 0.691). This also applies to the seven most common IR (Fig. 2) (0.150 ≤ *p ≤* 0.844). Accordingly, there is no difference in the assessment of acute diseases between parents who used and did not use the most common OR (family member or friend: *p =* 0.809, doctor: *p =* 0.433, pharmacist: *p =* 0.649). The education level of mothers has no influence on their assessment of acute diseases (*p =* 0.724). However, a higher education level of fathers is associated with a more serious estimation of the disease severity (*p =* 0.024). The very opposite applies to fathers with a lower education level.

Most of the parents attending the out-patient clinic with a child suffering from an acute illness and having used any kind of child health resource to inform themselves about it (*n =* 58), tend to feel no change in disease severity assessment (82.8 %/*n =* 48). Only 13.8 % (*n =* 8) rate the disease as more severe and 3.4 % (*n =* 2) as less severe, after having used any information resource (Fig. 1). For internet users the proportions are 79.2 % (unchanged, *n =* 38), 16.7 % (more severe, *n =* 8) and 4.2 % (less severe, *n =* 2) (Fig. 1). No statistically significant difference between change in disease severity assessment of parents with and without IUC could be proven (*p =* 0.999). The same applies to the seven most common IR (Fig. 2) (0.335 ≤ *p ≤* 0.886) and the most common non-internet CHI resources (family member or friend: *p =* 0.888, doctor: *p =* 0.239, pharmacist: *p =* 0.160). The education level of mothers (*p =* 0.261) and fathers (*p =* 1.0) has no influence on CA.

The tolerance values of the respective linear regression models were all ≥ 0.721 and, hence, no multicollinearity of the variables IUC and the three most common OR was assumed when determining their influence on IL, DA and CA.

### Health seeking before the appointment and sources of information

Of all parents attending the outpatient clinic, 28.7 % (143/499) inform themselves on the reason for consultation using any source of information. Twenty-one percent (105/499) use the internet for this purpose (= IUC) (see also [9]) and 15.7 % (78/498) non-web-based sources. Of the parents attending with a child suffering from an acute disease, 32.2 % (89/276) use any source of information, 24.3 % (67/276) use the internet and 17.5 % (48/274) use non-web-based sources to obtain information on this specific acute disease before the appointment.

Before attending the out-patient clinic, parents who use online child health information resources most likely search websites run by doctors (61.3 %), the FVPMC homepage (www.kinderarzt.at) (56.3 %), Google (40 %), Wikipedia (32.5 %), health advisory services provided by doctors (28.7 %), health portals (21.3 %) and health forums and communities (18.8 %) (Fig. 2). Eighty parents answered this item. Multiple answers were possible. All IR are shown in Fig. 2.

Analogously, before attending with an acutely ill child, parents most likely search websites run by doctors (58.8 %), the FVPMC homepage (www.kinderarzt.at) (56.9 %), Google (41.2 %), Wikipedia (33.3 %) and health advisory services provided by doctors (29.4 %), health portals (19.6 %) and health forums and communities (17.6 %). Proportions of parents using the remaining IR do not exceed 9.8 % and are not mentioned here. Fifty-one of the parents attending with an acutely ill child answered this item. Multiple answers were possible.

**Fig. 3:**
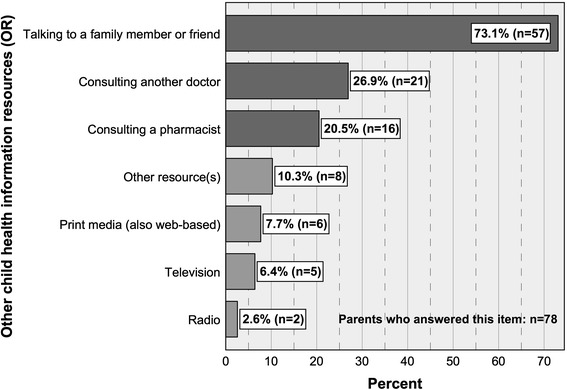
Other child health information resources (OR) with percentages of users and numbers of cases

Almost three out of four parents (73.1 %) who make use of other child health information resources consult a family member or friend. This portion is followed by parents who talk to another doctor (26.9 %) and parents who consult a pharmacist (20.5 %) before attending the pediatric out-patient clinic (Fig. 3). Seventy-eight parents answered this item. Multiple answers were possible. All OR are shown in Fig. 3.

Corresponding results were obtained for parents attending with an acutely ill child: of these, 70.8 % consult a family member or friend, 22.9 % talk to another doctor and also 22.9 % consult a pharmacist. Proportions of parents using the remaining IR do not exceed 10.4 % and are not mentioned here. Forty-eight parents, visiting the outpatient clinic with an acutely ill child, answered this item. Multiple answers were possible.

### Internet use (IUC) and influencing factors (IF)

**Fig. 4:**
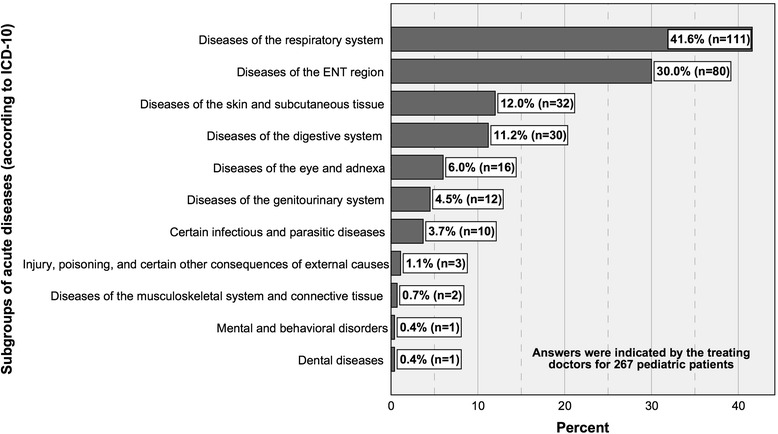
Subgroups of acute diseases (according to ICD-10) indicated by the treating doctors with percentages of sick children and numbers of cases. Subgroups that were not observed were excluded from the diagram (Table 1)

No statistically significant connection was identified between IUC and the sex of parents, implying that mothers and fathers do not differ in terms of their IUC (*p =* 0.767). Between IUC of older and younger parents no statistically significant difference could be proven (*p =* 0.616). However, there is a statistically significant difference between IUC of higher and less educated accompanying mothers (U = 9849.500; Z = −2.244; *p =* 0.025). In the sense that higher educated mothers are more likely to search the web for information on the reason for consultation than less educated mothers. According to this, a statistically significant difference between IUC of higher and less educated fathers was identified (U = 385.500; Z = −2.083; *p =* 0.037). IUC and the sex of children are not connected statistically, which implies that parents of girls and boys do not differ in terms of IUC (*p =* 0.912). There is a statistically significant difference between parents of younger and older pediatric patients regarding their IUC (*p =* 0.012). These findings indicate that parents of younger children are more likely to use the internet in order to obtain information on the reason for consultation. Parents consulting the pediatrician due to an acute disease are also more likely to use the internet (*p =* 0.046) (previously reported in [9]), although there are no specific subgroups of illnesses (Fig. 4) that predispose to a higher IUC (0.071 ≤ *p ≤* 1.0). Parents attending due to a follow-up visit after an acute disease of their child are less likely to use the web (*p =* 0.018). IUC shows no statistically significant connections to other reasons for consultation (0.183 ≤ *p ≤* 1.0). Parents who use the internet to obtain information on the reason for consultation are also more likely to talk to a family member or friend (p < 0.01) or another doctor (*p* < 0.01) for this purpose. Consulting a pharmacist is not statistically linked to IUC (*p =* 0.054).

## Discussion

### Summary of important results

Parents rate their knowledge in terms of the reason for consultation predominantly as good or average. Acute diseases of their children are rather estimated to be mild or moderate and most of the parents feel no change in this assessment after having used any source of information. The respective proportions do not deviate substantially, if determined for internet users only. Parents do not differ with respect to IL, DA and CA, whether they use the internet prior to their visit to the pediatrician or not. This also applies to the seven most common IR. Parents who use the most common OR display no different IL, DA and CA than those who do not use these. The lower the education level of mothers, the lower they estimate their level of knowledge in terms of the reason for consultation. Acute diseases of children are more likely to be assessed as mild by less educated fathers. More than one out of four parents inform themselves on the reason for consultation ahead of the appointment. More than one out of five use the internet for this purpose (= IUC) (see also [9]) and more than one out of seven use non-web-based sources. Interestingly, websites run by doctors and the outpatient clinic’s homepage are used more frequently than Google and Wikipedia in conjunction with the appointment. Health portals, health forums and communities, are less commonly used but still relevant to parents. Nearly three in four parents making use of OR talk to a family member or friend. Approximately one in four talk to another doctor and one in five consult a pharmacist. The proportions of parents using the internet or other sources of information, certain IR or OR, do not differ substantially between all parents and those attending with an acutely ill child. The order of most common IR and OR is consistent between these groups. IUC is dependent on the age of pediatric patients, the education level of mothers and fathers and the reason for consultation. Parents who use certain OR are also more likely to use the internet before the appointment.

## Conclusions

The primary results of this investigation, corresponding to our primary objectives, allow us to answer the leading questions of this research article. Internet use of parents before attending a general pediatric outpatient clinic does not change their subjective information level and assessment of acute diseases.

It does not conform to our expectations that even more parents indicate their information level to be good (50.6 %) than to be average (46.7 %), and that merely 2.7 % of parents feel insufficiently informed before attending the outpatient clinic. Before the evolution of the internet, the respective results might have differed substantially, as health information can now be accessed by many people worldwide. With regards to online health seekers, our expectations are rather met (good: 42.7 %, average: 55.3 %, insufficient: 1.9 %), but, interestingly, most of the internet users rate their knowledge as average.

The use of web-based and non-web-based child health information resources does not exert an influence on the self-estimated information level of parents with regard to the reason for consultation. Accordingly, the majority of the parents estimated their knowledge as good or average, regardless of whether child health information resources were used or not. However, the validity of these findings is limited, as the assessment of the state of knowledge was purely subjective. It would be worth investigating, if these results could be objectified by the use of an evaluation through professionals (e.g., test, interview, etc.), or if this would produce different or even contradictory results. Such an approach would inevitably involve considerable effort.

Against the common perception that health information found on the internet might fuel panic-mongering, we could not determine a link between IUC and the assessment of acute diseases (DA and CA); which is also valid for the use of the seven most common IR and the use of OR. Frequently used IR like Google, Wikipedia and interactive online resources are often said to irritate or even panic health seekers. We could not substantiate this common believe with our data, although these findings have to be interpreted cautiously (see limitations). The overwhelming majority of parents (196 of 197) who attended with an acutely ill child rate the disease to be either mild (58.4 %/*n =* 115) or moderate (41.1 %/*n =* 81). Merely one parent (0.5 %) rated the disease to be severe. With regard to online health seekers, the proportions are distributed similarly (54.9 %, 45.1 %, 0 %). Although predominately assessed by medical laymen and not health care professionals, the low frequency of 0.5 % (and 0 %, respectively) is probably owed to the fact that our investigation was conducted at a general pediatric outpatient clinic and not a pediatric emergency department. The high percentages of parents who feel no change in the estimation of the disease severity (82.8 % and 79.2 %) indicate that the use of any CHI resource, and the internet in particular, is not very likely to change the assessment of acute diseases. This supports the aforementioned results, that DA and CA are neither influenced by IUC, nor by use of the seven most common IR. It would be worthwhile to gain a better understanding of the emotional and behavioral consequences for parents arising from the subjective estimation of disease severity.

We approached the aspect of health literacy by identifying connections between the education level of parents and IL, DA and CA. Thereby we could prove that higher educated mothers tend to rate their information level as better, whereas less educated mothers are more likely to estimate their knowledge as inferior. It is conceivable that this is not only owed to a lower level of health literacy but also a lack of quality low-threshold online resources. In addition, we found that acute diseases of children are more likely to be rated as mild by less educated fathers and, on the contrary, that a higher education level of fathers is associated with a more serious assessment. These aforementioned aspects regarding health literacy of parents highlight the importance of gendering in this context. The reasons causing parents to rate their information level and acute diseases of their children in a certain way need to be addressed in future studies.

Of all parents attending the outpatient clinic, 28.7 % inform themselves on the reason for consultation ahead of the appointment. Twenty-one percent use the internet for this purpose (= IUC) (see also [9]) and 15.7 % non-web-based sources. These findings prove the importance of health information seeking for parents before visiting a health care facility and the prominent role of the internet in this context.

The most common IR, used before attending the general pediatric out-patient clinic, are websites run by doctors (61.3 %), the FVPMC homepage (56.3 %), Google (40 %), Wikipedia (32.5 %), health advisory services provided by doctors (28.7 %), health portals (21.3 %) and health forums and communities (18.8 %). These results prove that there is a strong demand for online resources provided by doctors, and in this context especially pediatricians, as the two most frequently used internet resources as well as online health advisory services belong to this group. These findings cause us to speculate that online child health information provided by doctors is widely trusted by parents in need for information on the reason for their consultation. We previously determined the three most common groups of websites generally used to obtain child health information (Google and Wikipedia, websites provided by doctors and online resources with user-generated health information) [9]. Interestingly, the most commonly accessed resources before attending the pediatric outpatient clinic also belong to these three groups. However, internet use before the appointment shifts the order of the most common IR. These results provide evidence for the importance of websites provided by doctors when searching for contents related to specific medical conditions or other specific medical topics (e.g., vaccinations, preventive check-ups, etc.).

By far most of the parents who utilize other resources of child health information in conjunction with the appointment talk to a family member or friend (73.1 %). This is probably owed to the low-threshold access to this OR. It is not surprising that also many parents talk to other doctors (26.9 %) or consult a pharmacist (20.5 %) to get another opinion ahead of their visit to the pediatrician.

The proportions of parents using the internet or other sources of information, certain IR or OR, show no remarkable difference when comparing all parents and those attending with an acutely ill child. The order of most common IR and OR is furthermore consistent between these groups.

IUC is dependent on the age of pediatric patients, the education level of mothers and fathers, the reason for consultation and the use of certain OR. Younger children, a higher level of education and acute diseases of children (also reported in [9]), predispose parents to use the internet in conjunction with the appointment.

It is evident, that there is a higher incidence of infections in young children, which is true in particular for children at kindergarten and elementary school. At that age, approximately eight to twelve infections per year are considered to be normal. Nevertheless, frequent infections often leave parents frustrated, which might result in a higher IUC. An increased rate of vaccinations and preventive check-ups, necessary in young children, might also explain this fact. This is in analogy to IUG, on which we already reported [9]. However, IUG is not dependent on the education level of parents, quite contrary to IUC. It would be worth knowing, if a higher literacy level is associated with a stronger interest in specific health topics and medical conditions of children and if other barriers exist that prevent lower-educated parents from searching the internet for this purpose. Former research results suggest that there is a causal relation between education level and internet health seeking behavior [21, 22]. We could furnish proof for this regarding IUC but not for IUG [9].

In terms of internet use to be informed about the reason for consultation, we previously determined that 21 % use the internet for this purpose. This is most probably the case with children having an acute disease [9], which is a plausible motive for parents to seek assurance. Parents visiting the pediatrician due to a follow-up visit after an acute disease are less likely to use the internet. This is also comprehensible, as in this case it seems probable that child health information has already been sought in conjunction with the previous appointment.

Parents who seek web-based information on a specific medical condition or topic before the appointment tend to talk to another doctor and consult a family member or friend more often for the same purpose. One explanation might be that online health seeking raises new questions that need to be addressed. It is furthermore conceivable that parents who display a stronger general interest in their child’s health, or even worry, are also more likely to get a second opinion. Another motive might also be that other sources of CHI seem more credible and are therefore used additionally to gain reassurance. These aspects are worthwhile investigating as part of future research.

Other IF like the sex of parents and children exert no influence on IUC; which is in line with our findings relating to parents and their IUG [9], although being contradictory to former research results relating to health seekers in general. These results substantiate our concept of a specific internet health seeking behavior of parents among health seekers in their entirety. Compared to IUG [9], the internet use before attending the pediatric outpatient clinic is not dependent on the age of parents. This finding raises the question, why younger parents are more likely to use the internet in general to seek CHI, although not displaying a higher propensity to use the web before an appointment. This provides a starting point for further research. Although not differing from IUG in all respects, the aforementioned findings relating to IUC provide indications that a distinction between IUG and IUC is necessary and reasonable; at least, when considering the internet health seeking behavior of parents.

All doctors dealing with parents of younger children, parents with a higher level of education and parents of children suffering from acute diseases, should be aware of their high internet use; in particular, when providing advice on how to find trustworthy IR and how to deal with the available contents appropriately. However, low-use groups must not be disregarded, as the motives and reasons for a less frequent use remain unclear. A lack of quality resources for parents of school children and adolescents as well as parents with a lower education level might be one reason for a lower use. Further investigations are needed to clarify the causes.

### Limitations

Being mindful of the limitations of questionnaire-based research, our data should be interpreted with caution. Reporting bias in our setting was not able to be controlled, as parents were asked to complete the questionnaires entirely on their own and were only given explanations in case of uncertainties. We merely obtained answers from those parents who were willing to complete the questionnaires and, therefore, we cannot be sure that our findings are applicable to all parents visiting the FVPMC. The study by definition selected parents who actually visited the outpatient clinic but excluded those who did not. The reasons that keep parents from attending and their potential relation to the internet health seeking behavior therefore remain unclear. This also applies to IL, DA and CA of parents who did not visit the outpatient clinic and to other factors – besides IUC and OR–with an influence on these variables. We did not ask non-users of CHI resources about their CA. Therefore, we had no control group for users of CHI resources regarding this variable. There was no item gathering the professional involvement or any other previous knowledge of parents in the field of medicine, which might have confounded the results regarding IL, DA and CA. We only asked parents if, but not when exactly, they had looked up information regarding the reason for consultation, assuming a close temporal relationship to the visit at our outpatient clinic. This might also have confounded the results pertaining to IL, DA and CA. The information level of parents was assessed subjectively on the part of parents. Therefore, no conclusions can be drawn on how IUC and the use of OR influence the objective knowledge of parents. Our data provide no evidence of actual disease severity, as it would be diagnosed by a pediatrician; and, consequently, no reliable conclusions can be drawn from these findings beyond the purely subjective assessment of parents.

## Abbreviations

CA: Change in disease severity assessment
CHI: Child health information
DA: Disease severity assessment
FVPMC: First Vienna Pediatric Medical Center
IF: Influencing factors with regard to IUC
IL: Information level regarding the reason for consultation
IR: Internet child health information resources
IUC: Internet use to be informed about the reason for consultation
IUG: General internet use to obtain child health information
OR: Other child health information resources
